# Addressing multimorbidity in Leprosy: A retrospective chart review from India

**DOI:** 10.1371/journal.pntd.0013503

**Published:** 2026-02-17

**Authors:** Joydeepa Darlong, Karthikeyan Govindasamy, Paramjit Gill

**Affiliations:** 1 The Leprosy Mission Trust India, New Delhi, India; 2 Warwick Centre for Global Health, University of Warwick, Coventry, United Kingdom; George Washington University Medical Center, UNITED STATES OF AMERICA

## Abstract

**Introduction:**

Leprosy remains a public health challenge in India, with many individuals continuing to experience physical disability, stigma, and psychosocial burden despite being cured. While co-occurrence of individual comorbidities such as diabetes and depression has been reported, the prevalence and patterns of multimorbidity among persons affected by leprosy remain poorly documented.

**Methods:**

We conducted a mixed-methods study across six tertiary referral hospitals of The Leprosy Mission in India during 2021–2022. Quantitative data were extracted from electronic medical records of 10,428 adults affected by leprosy, including clinical details, random blood sugar, haemoglobin, cataract assessment, and wellbeing status measured using the WHO-5 index. Multimorbidity was defined as the presence of ≥2 conditions in addition to leprosy. Logistic regression was used to identify associated factors. Qualitative data were collected through focus group discussions (FGDs) with 16 persons affected by leprosy and 14 healthcare providers and analyzed thematically.

**Results:**

Leprosy alone was present in 81.9% of participants, while 16.4% had one additional condition, and 1.7% experienced multimorbidity. Diabetes was the most frequent comorbidity (9.3%), followed by poor wellbeing (5.6%) and cataract (1.5%). Increasing age, male gender, and disability were significantly associated with multimorbidity. FGDs revealed limited awareness of multimorbidity, challenges in accessing integrated care, financial barriers, and absence of flexible guidelines for management.

**Discussion & conclusion:**

Although relatively uncommon (1.7%), multimorbidity among persons with leprosy poses substantial challenges. Diabetes, mental health concerns, and malnutrition highlight the need for integrated, person-centred care models. Strengthening primary healthcare, enhancing screening, and developing multimorbidity-sensitive guidelines are essential to improve outcomes, reduce stigma, and promote holistic wellbeing in this marginalized population.

## Author summary

Leprosy is a long standing disease that affects the skin, nerves, and sometimes permanent disability. Overall, the health and well-being of the individual gets compromised. In this study of more than 10,000 people with leprosy, most had leprosy alone, but some also had other health problems such as diabetes, cataracts, or poor mental wellbeing. A small number had more than one of these problems at the same time (called multimorbidity). These findings show that people with leprosy often need to be provided care for several health issues at once, not just leprosy. Treating leprosy in a more holistic way — addressing all physical conditions, giving appropriate health education and information to manage multiple conditions simultaneously and mental health — will help people live healthier and fuller lives.

## Introduction

Leprosy is prevalent in India with unquantified number of people cured of leprosy suffering from permanent disabilities [1]. Leprosy affected individuals suffer not only from the physical manifestations but also from stigma and impaired wellbeing [2]. Studies have shown that the proportion of people affected by leprosy have another health condition as well [3] such as depression and anxiety [4–6] diabetes [7,8], or neuropathic pain [9,10]. While the co-occurrence of individual comorbidities has been documented, there is dearth of evidence about the prevalence and [11,12]—among people living with leprosy.

The burden of multimorbidity is well recognized in chronic diseases such as tuberculosis, diabetes, and other neglected tropical diseases, but its extent in leprosy remains underexplored. Given that leprosy disproportionately affects underprivileged populations, often with poor nutritional status, there is reason to suspect a heightened vulnerability to coexisting chronic conditions.

This study documents the prevalence and pattern of multimorbidity among people affected by leprosy.

## Methods

### Ethics statement

The research was performed in accordance with the Declaration of Helsinki for Human Research of the World Medical Association and approval has been granted by The Leprosy Mission Trust India Ethics committee (Approval number: C-071/ TLMTI EC/24), DHR EC-Registered no. - EC/NEW/INST/2024/4670.

### Study setting

A mixed methods approach, encompassing chart review and focus group discussions (FGD), was undertaken in six TLM Hospitals, two in Uttar Pradesh, two in West Bengal, one in Delhi and one in Tamil Nadu. The Leprosy Mission Hospitals are tertiary referral centres for leprosy, with expertise in diagnosing and managing the disease and its related complications. In addition to routine clinical data specific for leprosy, a routine set of laboratory investigations and individuals’ wellbeing status are recorded for all patients registered for treatment. A common Health Information System has been in use since 2008 across all hospitals of The Leprosy Mission with a unique identification number for patients coming for treatment.

Chart review involved cross-sectional analysis of secondary data extracted from the electronic medical records of patients affected by leprosy who attended TTLM Hospitals. Clinical assessment data collected at the time of leprosy diagnosis or during the first three visits were extracted for the analysis. All TLM hospitals systematically maintained electronic health records that included demographic and clinical information, disability status, and standardized assessments of activity limitation and social participation. Subjective wellbeing was assessed using the WHO-5 Wellbeing Index (ref) at baseline and during follow-up visits. Each patient record was stored under a unique hospital identification number.

Data included individuals aged 18 years and above who were registered for leprosy treatment or treatment of its complications at the 6 selected TLM hospitals across 4 states in India during 2021 and 2022. Patients with missing data on key variables were excluded from the final analysis.

### Data extraction

De-identified data on WHO-5 wellbeing scores, hemoglobin (Hb), cataract and random blood sugar (RBS) levels were extracted, along with relevant clinical, demographic details, including type of leprosy (PB/MB), bacteriological index and disability status as WHO disability grade These routine laboratory values are usually collected prior to treatment initiation or when required, enabling identification of coexisting morbidities. Data was extracted by a software company that designed and maintains the Health Information System of TLM Hospitals. Data was extracted into Excel spreadsheets and checked for missing data and extreme values using validation rules.

The WHO-5 Wellbeing Index, validated for use in the Indian context, was used. It comprises five items scored from 0 (none of the time) to 5 (all the time). The raw score ranges from 0 to 25, which was converted to a percentage score (raw score × 4). A percentage score of ≤50 was considered indicative of low wellbeing.

India-specific cut-off values were used to identify elevated blood glucose levels as indicators of presence of diabetes. The presence of cataract is assessed using standard clinical assessment of eye.

FGD explored the perceived impact of multimorbidity. One FGD was conducted with persons affected by leprosy and the other with healthcare providers. Sampling was purposive to ensure representation of diverse experiences across different stages of disease progression among those affected. The health professionals comprised medical officers who were purposively selected based on having at least two years’ experience in managing leprosy in their clinics.

Participatory research principles were used for the FGDs, aiming to co-develop a causal model illustrating the relationship between multimorbidity, psychosocial challenges, and wellbeing (S1 File). Discussions were audio-recorded, transcribed verbatim in English, and supplemented by field notes taken by independent researchers to capture non-verbal cues and contextual information.

### Analysis plan

Descriptive statistics were used to describe the study participants’ demographic and clinical details. Prevalence of individual each condition was presented as percentage of patients with either diabetes, poor wellbeing or cataract. Patterns of multimorbidity was presented as leprosy plus one, two or three conditions in individual patients. Factors associated with multimorbidity was assessed using multiple logistic regression analysis. Age, gender, treatment status (as newly diagnosed, on treatment, completed treatment and relapse), bacteriological index and disability status WHO disability grade was included as explanatory variables. Data was analysed using the R statistical program.

Qualitative data was analysed thematically using NVivo software. A coding framework was developed inductively from the transcripts. Emerging themes and subthemes were organized into matrices to identify key patterns and relationships. These insights formed the basis for constructing a causal model and informed the development of a theory of change for future intervention study to manage multimorbidity and wellbeing in persons affected by leprosy.

## Results

During the year 2021–2022, a total of 10,441 patients were registered either as newly diagnosed or on treatment (MDT) or those who had completed treatment visiting hospital for some complications. Of them, 13 patients were excluded due to illogical values in the extracted data. The details of the remaining 10,428 patients recorded at the first three visits were included in the analysis. The demographic and clinical details of the patient records included in the analysis is shown in Table 1.

**Table 1:** Demographic and clinical profile of the study participants.

Characteristics	Categories	Total (n = 10428)
Age	<15 years	424 (4.1%)
	15 – 30 years	2815 (27.0%)
	31 – 45 years	2975 (28.5%)
	46 – 60 years	2677 (25.7%)
	> 60 years	1537 (14.7%)
Gender	Male	7301 (70.0%)
	Female	3127 (30.0%)
Treatment status	Never treated	3887 (37.3%)
	Under treatment*	1679 (16.1%)
	Care after cure	4820 (46.3%)
	Relapse	21 (0.2%)
	Missing	21
Type of leprosy	MB	4504 (94.2%)
	PB	279 (5.8%)
	Missing	5645
Delay at diagnosis	< 1 year	1222 (21.5%)
	1 – 2 years	1964 (34.4%)
	3 – 4 years	715 (12.5%)
	> 4 years	1806 (31.6%)
	Missing	4721
Bacteriological Index	0	4104 (82.5%)
	1-2	319 (6.5%)
	3 and above	557 (11.0%)
	Missing	5448
WHO disability grade	0	3625 (42.5%)
	1	1503 (17.7%)
	2	3415 (39.8%)
	Missing	1885

* Currently under treatment, partially treated elsewhere currently under treatment, restarted treatment.

The percentage of children was 4.1% (424), and 14.7% were more than 60 years old. The majority,70% were male. Among those included, 46.3% completed multi-drug therapy, followed by those who had never been treated (37.3%), those under treatment (16.1%), and 0.2% [13] patients who relapsed. MB type of leprosy was the majority, 4504 (94.2%), and 279 (5.8%) had PB type of leprosy. Of those with a recorded duration of disease before diagnosis; 44% had more than 2 years of duration. Of those with BI recorded, 17.5% had a positive BI, and 5,448 had missing BI details. Of those with WHO disability grade recorded, 57.5% had either grade 1 or 2 disability.

The pattern of multimorbidity among people affected by leprosy is shown in Table 2. Among 10,428 persons with leprosy, the majority had leprosy alone (n = 8,542; 81.9%). 1,713 individuals (16.4%) were affected by leprosy with one additional condition, while 167 (1.6%) had two additional conditions. Only a very small proportion (n = 6; 0.1%) experienced complex multimorbidity, defined as leprosy with three or more conditions.

**Table 2:** Patterns of multimorbidity.

Patterns	n (%)
Leprosy alone	8542 (81.9%)
Leprosy + 1	1713 (16.4%)
Leprosy + 2	167 (1.6%)
Leprosy + 3 or more	6 (0.1%)
Total	10428

The prevalence of a combination of different multimorbidity is shown in Table 3.

**Table 3:** Prevalence of multimorbidity.

Prevalence	n (%)
Leprosy alone	8542 (81.9%)
Leprosy + poor inner wellbeing	579 (5.6%)
Leprosy + cataract*	160 (1.5%)
Leprosy + diabetes	974 (9.3%)
Leprosy + diabetes + cataract	51 (0.5%)
Leprosy + diabetes + poor inner wellbeing	99 (0.9%)
Leprosy + poor inner wellbeing + cataract	17 (0.2%)
Leprosy + diabetes + Poor inner wellbeing + cataract	6 (0.1%)

* Cataract due to prolonged steroid therapy or age-related.

Leprosy plus one comorbid condition was present in 16.4% and remaining had two or more multimorbidity. Multimorbidity was observed in 173 individuals, giving a prevalence of 1.7%. The most frequent multimorbidity combinations included leprosy with diabetes and poor inner wellbeing (0.9%), followed by leprosy with diabetes and cataract (0.5%).

Factors associated with multimorbidity is shown in the Table 4. Increasing age, males, and the presence of disability were associated with multimorbidity. The association remained the same in the multivariate analysis.

**Table 4:** Factors associated with multimorbidity (n = 4066).

Factors	Categories	Multimorbidity	Unadjusted odds ratio	Adjusted odds ratio
**Age**	< 15 years	11/179 (6.1%)	**Ref**	**Ref**
	15 – 30 years	180/1071 (16.8%)	3.1 (1.7 – 6.1)	2.5 (1.4 – 5.0)
	31 – 45 years	293/1138 (25.7%)	5.3 (3.0 – 10.5)	4.3 (2.4 – 8.5)
	46 – 60 years	355/1064 (33.4%)	7.6 (4.3 – 15.1)	5.7 (3.2 – 11.4)
	> 60 years	205/614 (33.4%)	7.7 (4.3 – 15.3)	5.4 (3.0 – 10.8)
**Gender**	Female	314/1360 (23.1%)	**Ref**	**Ref**
	Male	730/2706 (27.0%)	1.2 (1.1 – 1.4)	1.1 (1.0 – 1.3)
**Treatment status**	Never treated	322/1388 (23.2%)	**Ref**	**Ref**
	Under treatment	145/541 (26.8%)	1.2 (1.0 – 1.5)	1.1 (0.8 – 1.3)
	Care after cure	576/2134 (27.0%)	1.2 (1.0 – 1.4)	1.0 (0.8 – 1.1)
	Relapse	1/3 (33.3%)	1.7 (0.1 – 17.3)	1.1 (0.0 – 12.0)
**Bacteriological index**	0	867/3417 (25.4%)	**Ref**	**Ref**
	1 - 2	64/224 (28.6%)	1.2 (0.9 – 1.6)	1.3 (0.9 – 1.8)
	>2	113/425 (26.6%)	1.1 (0.8 – 1.3)	1.2 (1.0 – 1.5)
**Disability**	0	317/1809 (17.5%)	**Ref**	**Ref**
	1 & 2	727/2257 (32.2%)	2.2 (1.9 – 2.6)	1.9 (1.6 – 2.2)

### Qualitative results

FGD with persons affected by leprosy included 16 individuals (12 male, 4 female), aged between 25 and 76 years with varying complications, including lepra reactions, disabilities, ulcers, diabetes, and hypertension.

**1. Awareness of Multimorbidity**

Participants were not aware that leprosy could coexist with multiple health conditions, even though some of them claimed to have more than one illness apart from leprosy. Some were surprised and worried, especially those recently diagnosed and not yet experiencing complications. One participant stated:

*“After knowing that leprosy can coexist with multiple diseases, this disease should not come to anyone.” (Participant 04, 46 M)*

**2. Perceived Impact of Multimorbidity on Daily Life**

Several participants reported that the coexistence of diseases had adversely affected their ability to work and fulfil family roles. One participant noted, *“I am the only earning member. After leprosy and deformities in the hands, I stopped working due to weakness.” (Participant 07, 54 M)*

More than half of the group mentioned that although their work capacity had declined, they continued to work in order to support their families. Fatigue, physical limitations, and fear of future complications were commonly voiced concerns. Participants expressed apprehension about the burden of taking multiple medications and coping with side effects: *“If I get multiple problems, do I have to take more medicines? It will be difficult to take all medicines for each problem.” (Participant 02, 41 M)*

**3. Access to Treatment**

Participants described fragmented and inconsistent access to care. While some preferred to seek care at district hospitals where they perceived services to be more comprehensive, many expressed a desire to receive care closer to home. Several participants reported first consulting local village doctors and only visiting higher-level facilities when symptoms persisted. They were uncertain whether comprehensive treatment for all conditions could be obtained at the primary care level. One participant with multiple morbidities stated, *“have leprosy, diabetes and recently diagnosed with hypertension. It is difficult for me to take all medicines together and follow the doctor’s advice”. (Participant 13, 65 M)*

**4. Availing Healthcare and Costs**

Logistical and financial barriers to accessing multiple health services were reported. Participants spoke of their desire to get treatment closer to home. While some expressed a preference for free government services, others acknowledged that these were often suboptimal, concerned about getting the correct treatment closer to home but remained the only viable option due to financial constraints. “*I don’t know if I will get treatment for all the problems (multiple morbidities) near my home in the block hospital (Community Health Centres)”. (Participant 05, 50 M)*

**5. Social and Psychosocial Impacts**

Participants, especially those with disabilities, felt challenged coping and were worried that addition of another chronic illness would burden them further. Worry and concern were the primary emotions expressed. There was confusion about where they could seek treatment if there were multiple conditions to be dealt with. The mental health impact of leprosy was unknown to them and they felt disturbed when they became aware. *“Feeling concerned that person with leprosy can develop mental health problems”.* Mental health complications were a source of fear and anxiety. *(Participant 01, 37 M)*

**FGD with health care providers**

A total of 14 healthcare providers (12 male, 2 female) participated in the FGD, with an age range of 25–62 years. Participants included medical officers (n = 5), rural health officers (n = 3), community health officers (n = 3), and non-medical supervisors (n = 3) and all of them were actively managing leprosy patients in their clinics.

**1. Recognizing and Experience of Multimorbidity**

Most healthcare providers acknowledged that multimorbidity was common in general practice, particularly among patients with non-communicable diseases (NCDs) such as diabetes and hypertension. However, the recognition of multimorbidity in the context of leprosy was less familiar. Several participants remarked that this was the first time they were consciously reflecting on multimorbidity in leprosy-affected individuals. One medical officer (participant) noted, *“Multimorbidity is not new, but in leprosy we are hearing (about it) for the first time.” (MO_ 03_male)*

While leprosy reactions and chronic complications were familiar clinical challenges, many providers had not considered co-existing chronic conditions among persons with leprosy, despite routinely treating them. One senior experienced leprosy health supervisor stated, *“In leprosy, some problems you can tackle, but not all. If patients develop reactions multiple times, how much medicine can we give? With multiple problems, it will be even more difficult.” (MO_ 06_male)*

**2. Health System issues**

Reduced drug availability, inadequate diagnostic capacity, and high patient load were commonly cited challenges. Many reported seeing 25–80 patients per day, leaving little time to explore complex health issues such as multimorbidity. One medical officer remarked, *“We do not have sufficient time to talk to patients in detail, so we miss the diagnosis of many problems.” (MO_ 08_male)*

Basic laboratory investigations required to confirm common comorbid conditions were often unavailable due to lack of reagents or equipment at primary health centre level.

**3. Uncoordinated Care and Continuity Challenges**

Care for persons with leprosy and multimorbidity provided was fragmented, with patients visiting multiple care providers across the health system. Migration for seasonal work was identified as a major barrier to treatment adherence. Patients often sought treatment in their home village but relocated before follow-up which disrupted the care for both leprosy and coexisting conditions.

Inadequacy of referral systems, inconsistent documentation, and lack of care coordination were barriers to effective management. One provider noted, *“Documentation is a central issue. As we do not see the same patient every time, it is difficult to track progress or manage problems effectively.” (MO_ 05_female)*

**4. Guideline Gaps and Clinical Confidence**

Existing guidelines—including those from the National Leprosy Eradication Programme (NLEP) and organizational protocols—were limited in scope and did not consider multimorbidity. Several medical professionals expressed uncertainty about deviating from disease-specific guidelines to accommodate complex cases. They acknowledged the need to use clinical discretion in the absence of clear guidance. As one provider put it, *“Although we do not have guidelines, we need to use clinical discretion to decide on management. However, we need some basic guidance on how to manage multiple problems in leprosy.” (MO_ 08_ male)*

Guidelines for multimorbidity were desired to be flexible, context-specific, and feasible within the constraints of Indian primary care settings. Many felt that guidelines developed in high-income countries were not applicable to their setting.

**5. Person-Centred Care and Outcome Expectations**

All agreed that the primary goal of managing multimorbidity should not be limited to disease-specific outcomes but should aim to improve overall functioning and quality of life. One participant stated, *“The outcome should be focused on the person, not specific to disease—for example, improvement in diabetes, hypertension, and leprosy together.” (DLO_15_Male).* Providers supported the need for shared decision-making, considering both clinical risks and financial burden, especially in the context of limited resources.

**6. Suggestions for Intervention and Improvement**

Participants recommended several measures to improve the management of multimorbidity in primary care settings. The key points mentioned were

- Developing flexible, locally relevant guidelines for multimorbidity management in leprosy.
- Include guidelines on managing multiple conditions in leprosy in the existing guidelines
- Strengthening documentation and continuity of care mechanisms.
- Enhancing screening and early identification of comorbid conditions during routine leprosy care.
- Allocating more consultation time to enable comprehensive patient assessments.
- Building awareness among healthcare providers and communities regarding multimorbidity and its impact.

The importance of provider–patient communication was repeatedly emphasized. One provider noted, *“Giving correct information to patients is very important if we have to tackle multiple problems in leprosy.” (DLO_15_Male)*

## Discussion

This study shows that the prevalence of multimorbidity (≥2 conditions in addition to leprosy) was 1.7%, with single comorbidity being the dominant pattern. Although comprehensive studies on multimorbidity in leprosy are lacking, existing reports highlight the complex challenges faced when residual morbidity from leprosy coexists with chronic conditions such as diabetes, depression, or lymphatic filariasis, each requiring ongoing self-care [14].

Diabetes emerged as the most frequent comorbidity in our cohort (9.3%), followed by poor inner wellbeing (5.6%) and cataract (1.5%). Nearly 1% had a combination of leprosy, diabetes, and poor inner wellbeing, while a small group (n = 6) experienced all three concurrently. Notably, we did not classify permanent disability as a comorbidity; if included, the prevalence of multimorbidity would exceed 50%. Previous studies have similarly highlighted the burden of diabetes among leprosy patients. Saraya et al. (2012) reported a high prevalence in Kuwait [7]and called for routine screening and integrated management. Antunes et al. pointed to the lack of formal glucose monitoring protocols during reactional episodes [15], and Papang et al (2009) confirmed that steroid-induced hyperglycaemia is common and underdiagnosed [16]. This is especially relevant in the context of glucocorticoid-induced diabetes mellitus (GIDM), as clinicians must balance glycaemic control with the need for steroids to manage type 2 reactions, which themselves can worsen ulcer outcomes.

Mental health is another under-recognized comorbidity. In our study, 5.6% reported poor inner wellbeing, with 0.9% experiencing this alongside diabetes. A cross-sectional study by Govindasamy et al. found 33% of participants had moderate to severe depression and 19% had moderate to severe anxiety, linked to disability, stigma, and social disadvantage [4]. Butlin similarly emphasized that multimorbidity in leprosy extends to disability, stigma, and psychological suffering, and advocated for person-centred models of care integrating mental health [3].

Although tuberculosis was not observed in our cohort, its coexistence with leprosy has been documented. Deivasigamani et al. (2024) described a complex case of leprosy with TB, HIV, and diabetes [17], and a systematic review confirmed the clinical importance of TB–leprosy coinfection [18]. Shared use of rifampicin in both diseases creates treatment dilemmas, as does prolonged corticosteroid use in ENL, which can increase susceptibility to TB [19].

Malnutrition also remains a significant concern in low-income settings where leprosy is prevalent. Studies report high levels of undernutrition among leprosy patients: Vaz Mario found 57% undernourished [20], Jindal reported 14% underweight with anaemia linked to multibacillary disease [21], and Velmurugan’s meta-analysis associated poor diet with increased risk [13]. Rao similarly found undernutrition (BMI < 18.5) more common in those with disability, across age and sex groups [22]. Malnutrition impairs immunity, delays healing, and worsens treatment outcomes, while leprosy-related disability and stigma exacerbate food insecurity, creating a vicious cycle.

Overall, living with leprosy and multimorbidity creates a compounded burden, with stigma, disability, and overlapping chronic conditions contributing to delayed diagnosis, fragmented care, higher costs, and worsening disability [3,4,6,22]. Health systems often remain ill-equipped, as guidelines are disease-centric and rarely address overlapping morbidities [3,15]. Weak diagnostic capacity, poor referral pathways, and fragmented service delivery further limit integrated care [22]. Strengthening primary health care, improving multimorbidity-sensitive guidelines, and investing in training and referral networks are essential to meet the complex needs of persons with leprosy and coexisting conditions.

### Strengths and limitations

As far as we are aware this is the first study to document Multimorbidity in persons affected by leprosy. A strength of this study is the inclusion of participants from six large, specialized leprosy hospitals with extensive expertise in managing leprosy and its associated complications. All hospitals use a common contexualized standard WHO approved protocol for managing leprosy. A large sample of 10,441 patients were seen over 2 years. However, studying also has several limitations. First, the diagnosis of diabetes was based on random blood sugar (RBS) cut-off values. Second, the recording of ICD codes for specific conditions was inconsistent across medical records. Third, the WHO-5 Wellbeing Index measures subjective well-being and does not provide a clinical diagnosis of mental health conditions. All this may have led to an overestimation of its prevalence. Additionally, participants with missing clinical data were excluded from the regression analysis, which may have affected the study findings. It is important to note that, as a retrospective chart review, the study was limited by the quality and completeness of the existing medical records.

## Conclusion

This study demonstrates that multimorbidity among persons affected by leprosy, though relatively uncommon at 1.7% in our cohort, presents a substantial clinical and public health challenge due to the complexity of coexisting conditions. Diabetes emerged as the most frequent comorbidity, underscoring the need for routine screening and integrated management of metabolic disorders during leprosy care, particularly in individuals receiving long-term corticosteroids. Mental health concerns, malnutrition, and the potential overlap with tuberculosis highlight the multidimensional nature of multimorbidity in leprosy, where biomedical, psychological, and social determinants intersect. Importantly, the exclusion of leprosy-related permanent disability from our definition suggests that the true burden of multimorbidity is considerably higher than reported.

The findings call for a shift from disease-specific, fragmented approaches to person-centred and integrated care models that address physical, mental, and social health needs concurrently. Strengthening primary health care, developing flexible multimorbidity-sensitive guidelines, and ensuring effective referral linkages are essential to improve outcomes for individuals with leprosy and multiple chronic conditions. Addressing these complex needs holistically will not only reduce disability and complications but also mitigate stigma, enhance quality of life, and advance equity in health systems serving marginalized populations.

## Supporting information

S1 File Qualitative part of the multimorbidity study. (DOCX)
