# Correction: Addressing multimorbidity in Leprosy: A retrospective chart review from India

**DOI:** 10.1371/journal.pntd.0014189

**Published:** 2026-04-06

**Authors:** Joydeepa Darlong, Karthikeyan Govindasamy, Paramjit Gill

The authors, Joydeepa Darlong and Paramjit Gill, should be noted as shared co-corresponding authors on this work. Paramjit Gill’s email address is: p.gill.1@warwick.ac.uk.
