# Supplementary material for: Addressing multimorbidity in Leprosy: A retrospective chart review from India
Source: PLoS Negl Trop Dis. 2026 Feb 17;20(2):e0013503. doi: 10.1371/journal.pntd.0013503 (PMC12923119; doi:10.1371/journal.pntd.0013503)
Supplement: S1 File — (DOCX) [file pntd.0013503.s001.docx]

**Qualitative part of the multimorbidity study**

**Purpose of the FGD – with communities of people affected by leprosy :** Engage with communities affected by leprosy to apprise them about the challenges in diagnosing and managing multimorbid conditions and the associated stigmatization and psychosocial impacts

**Participants characteristics**

Number of participants = 16 (Male = 12, Female = 4)

Age range = 25 years – 76 years

Leprosy related complications among FGD participants = Lepra reactions, leprosy-related disabilities, Ulcer, Diabetes, Hypertension

**Points for the discussion**

1. Prevalence of multimorbidity
   1. Have you been diagnosed with any other illness, other than leprosy?
   2. What is your response to the presence of “more than one disease“ multimorbidity among people affected by leprosy
   3. What does this multiple disease mean to you
   4. What is your experience – ask those with disability – How has living with more than one disease impacted your activities of daily living.
   5. Where is treatment available for these conditions? Disability, mental health problems, diabetes, hypertension – where do you go for each disease ? All solutions under one roof ? How many referrals have you faced ?
   6. Where do you think is a best place to get the treatment for all these multiple conditions – SCs, PHCs, CHCs or DHs, NGO ? Private hospitals ?
   7. How do you manage therapy for multiple conditions – missing medications , any side effects of taking multiple medicines ?
   8. Self-management – support system – who brings you to the hospital, helps you at home ?
   9. Spending money to visit various facilities ? Prefer free places even if you know they are not optimum?
   10. How has the presence of multiple diseases affected your role in the family, as a breadwinner, housewife, etc. Are you able to work as before, QOL as before

FGD with patients notes

At the start of the FGD, patients were informed about the available evidence on the presence of multimorbidity based on the secondary data. Key points of patients' responses are listed below.

Initial response

- Feeling worried to know that a person with leprosy can suffer from so many diseases
- I was diagnosed with leprosy a few months before, and has not experienced any problems so far.
- After knowing that leprosy can co exist with multiple disease, this disease should not come to anyone
- Feeling concerned that person with leprosy can develop mental health problems
- I find it challenging to live with disabilities, worried if I develop more problems due to leprosy (multiple morbidities)

Impact of the multimorbidity

- I am the only earning member. After leprosy and deformities in the hands I stopped working due to weakness in hands
- More than half of the group participants have said that their ability to work has reduced, but managed to meet the work demand to continue to earn money
- I don’t have enough strength to work with multiple problems.
- If I get multiple problems (morbidities), do I have to take more medicines? It will be difficult to take all medicines for each problem

Treatment for the multimorbidity

- I don’t know if I will get treatment for all the problems (multiple morbidities) near my home in the block hospital (Community Health Centres)
- I will visit the district hospital for all the diseases (multiple morbidities) for treatment
- I prefer to get treated for all my problems near to my home
- I will visit local doctor in the village first, then if the problem is not resolved, I will visit the district hospital
- I have leprosy, diabetes and recently diagnosed with hypertension. It is difficult for me to take all medicines together and follow the doctor's advice

**Purpose of the FGDs with health care providers:** FGD with healthcare providers to elicit their views on multimorbidity and how to address it

**Participants characteristics**

Number of participants = 14 (male = 12, female = 2)

Age range = 25 – 62 years

Designations: Medical officers = 5, Rural health officers = 3, Community health officers = 3, Non-medical supervisors = 3)

**Points for the discussion**

1. Prevalence of multimorbidity
   1. What is your first response to the extent of multimorbidity
   2. How do you think we can address
   3. What difficulties you anticipate
   4. Do you have sufficient time during the consultation to address multimorbidity - to explain risks associated with medications
   5. What do you think should be the outcome – improved function and quality of or disease specific outcomes
2. With the current structure of primary care – can we respond to the needs of patients with multimorbidity
   1. Fragmented care
   2. Specialists and MOs in the PHCs and CHCs
   3. When to refer and take over
3. Inadequacy of guidelines – NLEP, TLMTI guidelines
   1. Often restricted to single disease, is it useful
   2. How valid those guidelines are for those with multimorbidity
   3. How confident you are when you modify care to meet patients with multimorbidity. By modifying deviating from the protocol
4. Patient centred care vs guidelines based care
   1. What are the challenges
   2. What are the facilitators – you are associated with patients for a long time
5. Shared decision making
   1. Taking decisions based on the risks associated with the conditions being treated
   2. Taking decisions based on the cost involved in investigations

**
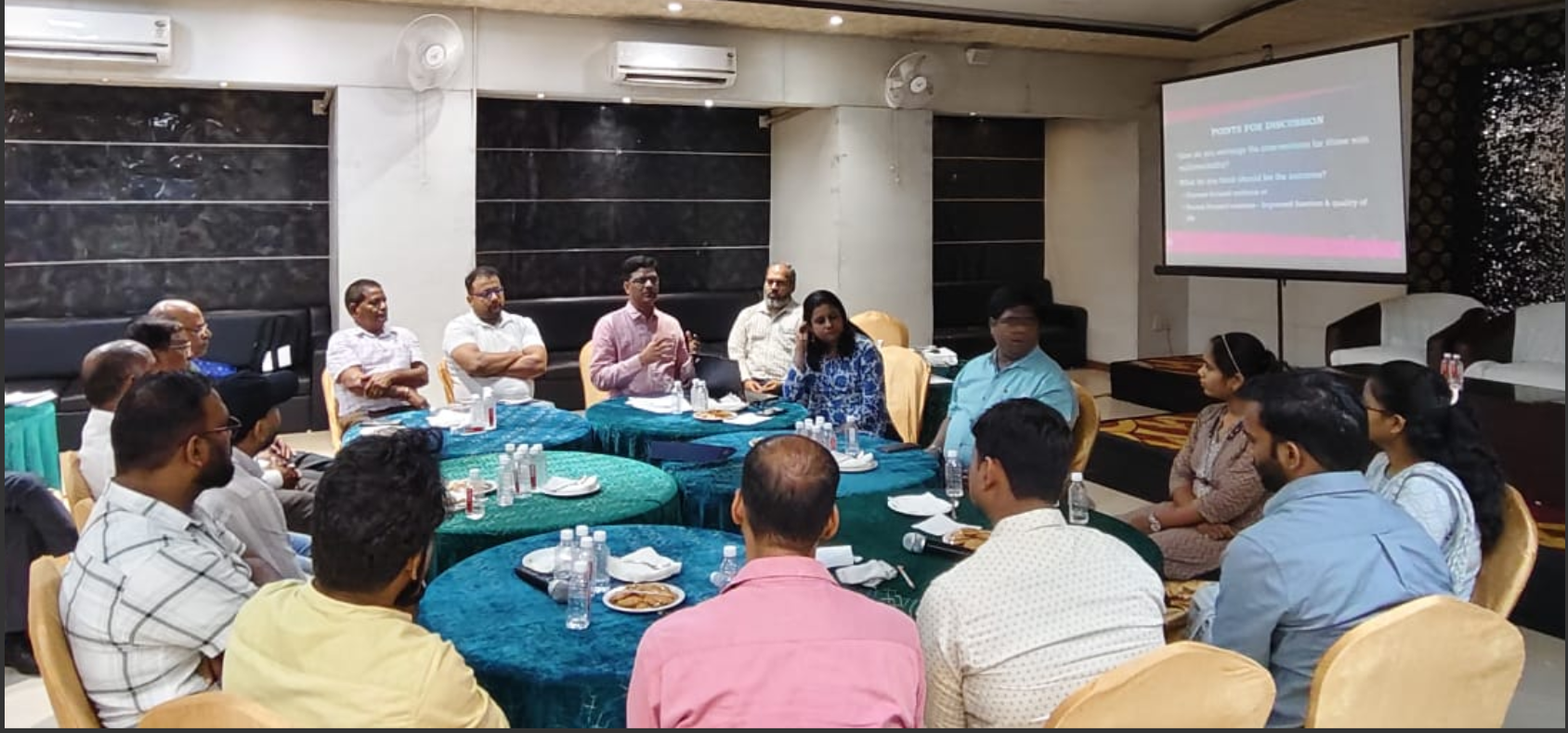
**

**
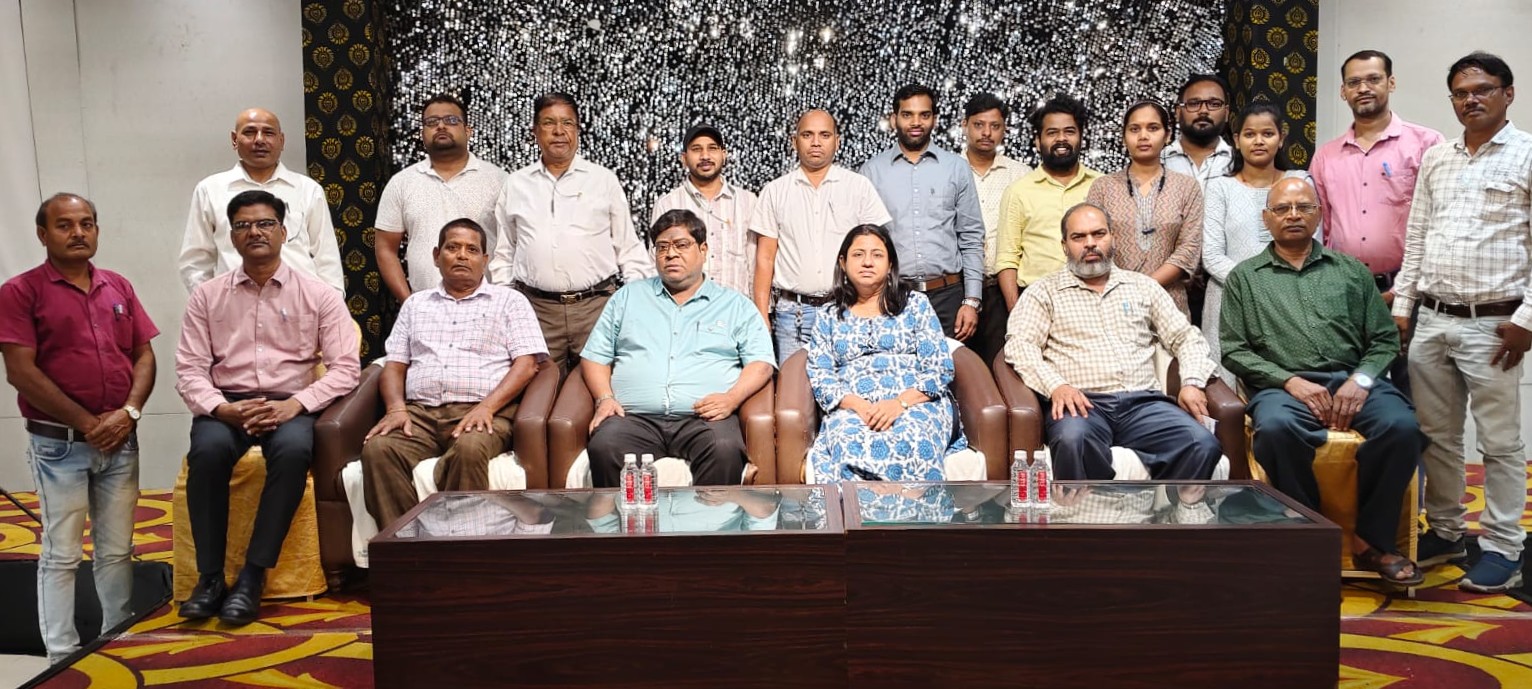
**

FGD started with the presentation on preliminary findings of the secondary data from the tertiary hospitals for leprosy

Key discussion points are given below taken from the transcript

How many diseases can be treated in the primary health centres

- Overall 70 to 80% of diseases can be treated in the PHCs
- Problems in PHCs is that not all the medicines are available
- Availability of some medicines are limited
- Patient don’t come for follow-up, migration is too much
- 25 to 80 patients per day
- We are managing the non-communicable disease better. None of our patients have complications
- To suspect leprosy is very difficult in the PHCs
- Some go to sub-centre (Health and wellness centre)
- Other than reactions patients complain of pain
- They also complain of mental health issues (man kharab and tenson and adbadana (anxiety))
- Some patients say that they are taking diabetes medication
- We did not see any patients with other than leprosy problem, but we have seen multimorbidities in the non-leprosy patients

From leprosy worker (NMS with 40 years of experience)

- In leprosy some problems you can tackle, but not all
- Patient can develop one or two times reactions, but many patients develop multiple time, how much medicine can we give. With multiple problems it will be even more difficult

About diagnosis and investigation of multimorbidity

- For leprosy, we don’t have any drugs for managing diabetes, hypertension. We have drugs only for leprosy only.
- When we see more problems in patients we refer to TLM hospital where all kind of treatment is available and patients are also satisfied
- Giving correct information to patient is very important if we have to tackle multiple problems in leprosy
- With one (leprosy) disease itself we have lot of problems in treatment, can’t imagine more than one disease in leprosy patients
- Multimorbidity is not new but in leprosy we are hearing first
- Medicines and tests in the PHCs are limited. Even if we want to rule out some disease, it is difficult, as we cannot person required tests in the PHCs
- Often we do not have reagents to perform tests in the health centres. Therefore, we cannot do test that we have to do to confirm other problems
- NMS says, for leprosy treatment, MOs often rely on NMS for diagnosis and treatment, then how we will manage the multiple problems
- Patients often stays in the village for few months, then goes to cities (migration) for livelihood. This affects treatment of leprosy and its complications
- Migration also affects the continuity of treatment and
- Patients sometime don’t want to do blood test
- Where all tests are available, such as district hospital, patient prefers to take treatment in the district hospital
- We do not have sufficient time to see patients, talk to patients in details, so we miss diagnosis of many problems

About management of multimorbidity

- Proper guideline on multimorbidity is not available
- For example, if patient has leprosy, also diagnosed with hypertension, there is no specific guideline
- Not all problems can be managed in the SC and PHC as tests and treatment is not available for confirmation of disease
- Sometimes we do not know immediately, when we see patients with multiple problems, so we can consult seniors before starting treatment
- Sometimes we do not have answer for all the problems of the patient, it is important to communicate clearly to patient come after few days for treatment
- Can we manage multimorbidity with the available resources in the PHCs and SCs? The answer is No, because we were unable to confirm the diagnosis immediately before starting the treatment. Without confirmation it is difficult to provide treatment
- Patient will have difficulty in understanding the treatment and continuation of follow-up
- Follow-up is essential for multimorbidity, but it is difficult in leprosy as they migrate to cities for livelihood
- Although we do not have guidelines, we need to use clinical discretion to decide on the management. However, we need some basic guidelines on how to manage multiple problems in leprosy.
- Guideline on multimorbidity to be flexible

Suggestions on management (intervention) of multimorbidity in primary care facility

- Documentation is a central issue. As we do not see the patients at every visit, it is difficult to ascertain the improvement and manage problems.
- We cannot follow guidelines developed in developed countries. We need specific guidelines that we can use in India
- Screening and follow-up are essential to identify problems early and manage early
- Need to have adequate time for talking to patient and taking history to suspect and diagnose multimorbidity
- We need to improve awareness of leprosy and multimorbidity in the community and among healthcare workers

Outcome – what needs to be the outcome of the management of multimorbidity

- The outcome should be focused on the person, not specific to disease. For example, improvement in diabetes, hypertension, and leprosy.
